# Systematic reviews in context: highlighting systematic reviews relevant to Africa in the Pan African Medical Journal

**DOI:** 10.11604/pamj.2016.24.180.10100

**Published:** 2016-06-30

**Authors:** Charles Shey Wiysonge, Raoul Kamadjeu, Landry Tsague

**Affiliations:** 1 Centre for Evidence-based Health Care, Faculty of Medicine and Health Sciences, Stellenbosch University, Cape Town, South Africa; 2 Cochrane South Africa, South African Medical Research Council, Cape Town, South Africa; 3 The Pan African Medical Journal, Center for Public Health Research and Information, Nairobi, Kenya

**Keywords:** Systematic reviews, Africa, evidence-informed research, evidence-informed policy, meta-analysis

## Abstract

Health research serves to answer questions concerning health and to accumulate facts (evidence) required to guide healthcare policy and practice. However, research designs vary and different types of healthcare questions are best answered by different study designs. For example, qualitative studies are best suited for answering questions about experiences and meaning; cross-sectional studies for questions concerning prevalence; cohort studies for questions regarding incidence and prognosis; and randomised controlled trials for questions on prevention and treatment. In each case, one study would rarely yield sufficient evidence on which to reliably base a healthcare decision. An unbiased and transparent summary of all existing studies on a given question (i.e. a systematic review) tells a better story than any one of the included studies taken separately. A systematic review enables producers and users of research to gauge what a new study has contributed to knowledge by setting the study’s findings in the context of all previous studies investigating the same question. It is therefore inappropriate to initiate a new study without first conducting a systematic review to find out what can be learnt from existing studies. There is nothing new in taking account of earlier studies in either the design or interpretation of new studies. For example, in the 18th century James Lind conducted a clinical trial followed by a systematic review of contemporary treatments for scurvy; which showed fruits to be an effective treatment for the disease. However, surveys of the peer-reviewed literature continue to provide empirical evidence that systematic reviews are seldom used in the design and interpretation of the findings of new studies. Such indifference to systematic reviews as a research function is unethical, unscientific, and uneconomical. Without systematic reviews, limited resources are very likely to be squandered on ill-conceived research and policies. In order to contribute in enhancing the value of research in Africa, the Pan African Medical Journal will start a new regular column that will highlight priority systematic reviews relevant to the continent.

## Commentary

When a patient survives a first heart attack, the heart may be so badly damaged that the patient will develop mechanical pump failure. A heart attack may also lead to irregularities in the heart rhythm because of heart muscle damage. Heart attack survivors who develop one or both of these two complications have a higher risk of death than those who do not [1]. In addition, when a patient survives a first heart attack, the chances are that he or she may suffer another heart attack; which will trigger a new set of electrical and mechanical complications. The work done by the left ventricle in pumping blood and the occurrence of heart rhythm irregularities are both increased by endogenous chemical substances known as catecholamines. The action of these substances on beta-adrenergic receptors, part of the sympathetic nervous system that mediates the ‘fight-or-flight’ response, is blocked by a class of drugs called beta-blockers [2]. On this basis, beta-blockers were proposed for use in people who had survived a heart attack in order to reduce the risk of future cardiac events. The first published randomised controlled trial for this indication was reported in 1972 [3]. A decade later, more trials had been done, but all trials were generally small in size and the individual results were confusing because none of the trial authors had taken a systematic account of previous similar trials. Had they done so, it would have been obvious that enough evidence had accumulated showing a beneficial effect of beta-blocker therapy for secondary prevention after a heart attack [4–6]. Following the publication in 1981 of the seventh study on the topic [7], a narrative review reported that there was still no convincing evidence that beta-blocker therapy led to long-term survival after heart attacks [8]. However, at that time there was already overwhelming evidence that giving a beta-blocker to heart attack survivors reduced the risk of future cardiac events [5, 6]. Yet, over the ensuing decade, trials continued to be done in which beta-blockers were compared to placebo or no treatment [9]. If a systematic review of existing trials had been done and the data pooled using an appropriate statistical method, the combined evidence would have shown that the conduct of further trials was unnecessary. The pointless trials, that continued to be undertaken, deprived half the trial participants of an effective treatment. We see here a failure to boost the significance of existing research and decrease waste in the conduct of new research, because of a failure to set each new study in the context of other relevant studies [10].

Narrative reviews, such as the one referred to above [8], are prone to multiple systematic errors (or biases). Authors of such reviews typically use subjective methods to collect and interpret existing data. One source of systematic error in narrative reviews is publication bias, which occurs when authors of a review only search for published studies. It is well documented that studies with statistically significant results are more likely to be published and more likely to be published in journals with high citation impact factors than studies with non-significant results [11]. Therefore, if a review of treatment effects only considers published studies, it is very likely to over-estimate the effectiveness of the treatment under consideration. Another common error in narrative reviews is language bias. For example, a study of German authors who had published different trials in both English and German language journals revealed that statistically significant results were more likely to be published in English [12]. Thus a review that searches only for English language publications is more likely to overestimate treatment effects. Narrative reviews may also ignore study design in the analysis of existing data. A systematic review was conducted to assess the effectiveness of vitamin supplementation in preventing cardiovascular disease [13]. The authors found a significant association between the use of vitamin supplements and a lower risk of cardiovascular mortality in cohort studies. However, randomised controlled trials of the same supplements failed to demonstrate a consistent effect. Beta-carotene, for example, significantly reduced cardiovascular mortality in cohort studies but significantly increased cardiovascular mortality in randomised controlled trials [13]. When studying the effects of interventions such as beta-carotene, randomised trials are better suited than cohort studies because they have the advantage of taking account of both known and unknown factors that may influence the risk of having the outcome (such as cardiovascular mortality in this case). Sources of bias, such as those discussed above, render the results of narrative reviews unreliable; thus the need for systematic reviews.

## Key characteristics of systematic reviews

The core element of a systematic review is an explicit attempt to collate the totality of relevant existing data on a clearly defined question [14]. A systematic review is characterised by a well-defined and focused question; pre-defined eligibility criteria for selecting studies; a comprehensive search strategy for identifying all potentially eligible studies; duplicate assessment of the risk of bias and extraction of data from included studies; an appropriate synthesis of data; and a complete presentation of the findings. Statistical aggregation (referred to as meta-analysis) may or may not be used to summarise data from studies included in a systematic review [15].

### Significance of systematic reviews

Without systematic reviews of previous research, ineffective or even harmful interventions may be used because they are thought to be effective and, conversely, effective interventions may be considered ineffective and withheld [16]. A systematic review should be the first step when defining questions for new research and when taking decisions about health care. When taking decisions about health care, there should be clear documentation of how relevant systematic reviews were identified and assessed for their quality, local applicability, potential impacts on equity, cost implications, and scaling-up considerations [17]. When systematic reviews are ignored, it is very likely that limited healthcare resources would be squandered on ill-conceived research and policies, and avoidable confusion would result from failure to set new research in the context of relevant existing research [18]. There is nothing new about systematic reviews. For example, in 1747 James Lind conducted a systematic review to assess the effects of various contemporary treatments for scurvy [1]. Another example is the emphasis of the importance of systematic reviews, to the British Association for the Advancement of Science, by Lord Rayleigh in 1884 [19].

### Highlighting systematic reviews in the Pan African Medical Journal

As shown in the preceding sections, a systematic review of existing research on a given topic paints a better picture of the topic than any one primary study taken separately. In order to increase the use of systematic reviews in Africa, the Pan African Medical Journal will start a new section called “Systematic reviews in context” that will highlight systematic reviews relevant to the continen**t.**

## Conclusion

Systematic reviews can help to identify what studies should be replicated, to avoid unnecessary duplication, and result in new studies that address deficits in previous ones. Despite the value of systematic reviews, there is empirical evidence showing that they continue to be ignored in both the design and the interpretation of findings of new research [19, 20]. This contempt for systematic reviews may occur because researchers are so preoccupied with a misguided philosophy of individual originality that they do not realise that research is a shared scientific activity, in which the community accomplishes more than the sum of the efforts of its members. This misguided philosophy should change, if the efforts of researchers are to yield maximum benefits to the wider community which continues to invest so much in research. We believe that the time for that change is now. This explains why the Pan African Medical Journal plans to regularly highlight systematic reviews relevant to Africa, with the hope that this would contribute to evidence-informed health research, policy, and practice on the continent.
